# VvRALF9–VvFER2 Module Mediates Grapevine Salt Stress Responses Through Hormone Signaling and Transcription Factor Regulation

**DOI:** 10.3390/plants15213396

**Published:** 2026-11-02

**Authors:** Kai Liu, Kaidi Li, Suya Shang, Huiping Liu, Yunning Pang, Xuzhe Zhang, Dongchen Wu, Fujun Zhang, Bo Li, Xiujie Li

**Affiliations:** 1 Shandong Academy of Grape, Shandong Academy of Agricultural Sciences, Jinan 250100, China; liukai2429@163.com (K.L.); lkd965322@163.com (K.L.); ssy12130@163.com (S.S.); 17862973825@163.com (H.L.); pangyunning@163.com (Y.P.); zhangxuzhe2002@163.com (X.Z.); 15235068482@163.com (D.W.); zhangfujun5279@163.com (F.Z.); sdtalibo@163.com (B.L.); 2 College of Life Sciences, Qingdao Agricultural University, Qingdao 266109, China; 3 College of Life Sciences, Shandong Agricultural University, Taian 271000, China

**Keywords:** grapevine, RALF gene family, salt stress, *VvRALF9*, *VvFER2*

## Abstract

Soil salinity severely limits grapevine yield and fruit quality; however, the mechanisms underlying salt tolerance remain poorly understood. Small secreted peptides, particularly rapid alkalinization factor (RALF) peptides, are key regulators of plant responses to environmental stress. However, a systematic analysis of the *RALF* gene family in grapevine (*Vitis vinifera* L.) has not been conducted. In this study, we identified 11 *VvRALFs* in the grapevine genome (PN_T2T) and characterized their physicochemical properties, structural features, and evolutionary relationships. Expression profiling revealed that *VvRALF9* was consistently upregulated in the roots of the salt-sensitive rootstock ‘Beta’ but downregulated in the salt-tolerant rootstock ‘3309C’. Silencing *VvRALF9* in the roots of grapevine rootstocks significantly enhanced their salt tolerance. Protein interaction assays, including yeast two-hybrid, split-LUC, and pull-down assays, identified VvFER2 as a potential receptor of VvRALF9. Overexpression of *VvFER2* in the roots of grapevine rootstocks also improved salt tolerance. Transcriptomic analysis of *VvRALF9*-RNAi roots revealed significant enrichment of the plant hormone signal transduction pathway, together with extensive changes in the expression of transcription factors and genes associated with multiple hormone signaling pathways. Together, these findings suggest that the VvRALF9–VvFER2 module is involved in the regulation of grapevine salt stress responses.

## 1. Introduction

Soil salinization is one of the most pressing environmental challenges facing global agriculture. Currently, saline soils account for approximately 7% of the Earth’s land area, covering nearly 1 billion hectares, and this area continues to expand [1,2]. Excessive soil salinity causes nutrient imbalance, oxidative damage, osmotic stress, and ion toxicity, thereby impairing plant growth, fruit quality, and crop productivity [2,3]. Grapevine (*Vitis vinifera* L.) is a perennial fruit crop of considerable economic importance and is widely cultivated worldwide for both fresh consumption and wine production [4]. Developing salt-tolerant grapevine cultivars and rootstocks using salt-tolerant germplasm represents an important strategy for sustaining grapevine production in mildly to moderately saline-alkaline soils. However, the molecular mechanisms underlying salt tolerance in grapevine remain incompletely understood, constraining the identification and effective utilization of salt tolerance-related genes and genetic resources in breeding programs [5].

Phytohormones are central regulators of plant intercellular communication [6], but increasing evidence indicates that small signaling peptides constitute another important class of intercellular signals involved in plant growth, development, and responses to environmental stress [7]. These peptides are generally secreted into the extracellular space and perceived by specific plasma membrane-localized receptors, thereby converting extracellular cues into intracellular signaling responses. Peptide–receptor signaling can therefore provide an important link between environmental perception and the physiological adjustments required for plant adaptation. In *Arabidopsis thaliana*, for example, the root-derived CLE25 peptide acts as a long-distance dehydration signal, and BAM1/BAM3-dependent signaling in leaves promotes abscisic acid (ABA) accumulation and stomatal closure under water-deficit conditions [8]. Such findings illustrate the important role of peptide-mediated cell-to-cell communication in coordinating plant responses to environmental stress.

Within this framework of cell-surface signaling, FERONIA (FER), a member of the *Catharanthus roseus* receptor-like kinase 1-like (CrRLK1L) family, acts as an important signaling hub linking extracellular cues to intracellular responses. FER participates in multiple processes associated with plant growth, cell-wall integrity, and environmental adaptation [9]. In *A. thaliana*, FER senses salt-induced perturbations in cell-wall integrity and activates cell-specific Ca^2+^ signaling, thereby facilitating root growth recovery under salinity [10]. Evidence from other species also supports a role for FER in salt adaptation. In apple (*Malus domestica*), *MdFER* is induced by NaCl and ABA; its overexpression enhances salt tolerance, whereas the suppression of *MdFER* increases salt sensitivity [11]. These findings suggest that FER-mediated cell-surface signaling represents an important component of the mechanisms by which plants perceive and respond to saline environments.

Rapid alkalinization factors (RALFs) are small cysteine-rich secreted peptides encoded by small open reading frames (ORFs). RALF was first identified during a peptide hormone screen of tobacco (*Nicotiana tabacum*) leaf proteins. In tobacco suspension cells, this peptide rapidly increases extracellular pH and activates intracellular mitogen-activated protein kinases, leading to its designation as “RALF” [12]. Subsequent studies have revealed diverse biological functions of RALF peptides across plant species. In *A. thaliana*, individual RALF members regulate processes ranging from primary root growth to pollen-tube integrity and reproductive signaling [13,14,15]. Several RALF members have also been implicated in plant responses to environmental stresses, indicating that this peptide family participates in the coordination of both developmental and stress-responsive processes [16]. Although the *RALF* gene family has been characterized in several plant species [13,17,18,19], it has not yet been systematically investigated in grapevine, and the specific VvRALF members involved in grapevine salt-stress responses remain largely unknown.

The biological functions of RALF peptides are frequently mediated through interactions with cell-surface receptors, particularly FER. Numerous studies have shown that secreted RALF peptides interact with the extracellular domain of FER and activate signaling cascades involved in plant growth, development, and environmental responses [20,21]. In *A. thaliana*, the leucine-rich repeat extensin (LRX)3/4/5–RALF22/23–FER module plays an important role in salt-stress signaling [22]. Increased extracellular Na^+^ promotes the dissociation of RALF22/23 from LRX3/4/5, accompanied by FER internalization, reduced FER signaling, ABA accumulation, and enhanced reactive oxygen species (ROS) production, ultimately increasing salt sensitivity [22]. Evidence from other species further supports the involvement of RALF-associated signaling in salinity responses. Disruption of *PpRALF3* enhances tolerance to NaCl and oxidative stress in *Physcomitrium patens* [23], whereas a conserved CqRALF–FER signaling system has been implicated in the salt-stress response of *Chenopodium quinoa* [24]. These findings indicate that RALF–FER signaling is closely associated with plant adaptation to salinity, although the specific regulatory mechanisms may differ among species. Plant responses to salinity are also coordinated by multiple phytohormone pathways, including ABA, jasmonic acid (JA), gibberellin (GA), salicylic acid (SA), and ethylene (ETH), together with stress-responsive transcriptional networks [25,26]. The established links between RALF–FER signaling and stress-associated hormonal responses therefore raise the possibility that hormone signaling and transcriptional regulation constitute important downstream components of RALF–FER-mediated salt responses.

However, whether a functional RALF–FER regulatory module operates in grapevine roots and how such a module is associated with downstream hormone signaling and transcriptional regulation remain unclear. To address these questions, we performed a genome-wide analysis of the *VvRALF* gene family and characterized its evolutionary, structural, and expression features. We further compared the salt-responsive expression patterns of *VvRALFs* in the salt-sensitive rootstock ‘Beta’ and the salt-tolerant rootstock ‘3309C’ to identify candidate genes associated with contrasting salt responses. *VvRALF*9 was subsequently selected for functional characterization in grapevine roots, and its interactions with grapevine FER proteins were investigated, followed by functional analysis of *VvFER2*. Finally, RNA-Seq analysis was performed to explore transcriptional changes associated with *VvRALF*9 suppression, with particular attention to hormone signaling pathways and stress-responsive transcription factors (TFs). Collectively, this study aimed to clarify the potential role of the VvRALF9–VvFER2 module in grapevine salt-stress responses and to provide new insights into the molecular mechanisms underlying salt adaptation in grapevine.

## 2. Results

### 2.1. Identification, Phylogenetic Relationship, and Synteny Analysis of VvRALFs

BLASTP searches were conducted using AtRALF protein sequences as references to identify potential VvRALFs [13]. Initially, 32 candidate proteins were detected, and the presence of the conserved RALF domain was subsequently confirmed (Figure S1). A final set of 11 *VvRALFs* was identified and designated as *VvRALF1* through VvRALF1*1* according to their chromosomal locations (Table 1). The molecular weight (MW) of these proteins spanned from 12.15 kDa (VvRALF1) to 14.60 kDa (VvRALF3), with protein lengths varying from 110 (VvRALF1) to 131 amino acids (VvRALF3). The isoelectric point (pI) spanned from 7.54 (VvRALF4) to 9.50 (VvRALF11).

**Figure 1:**
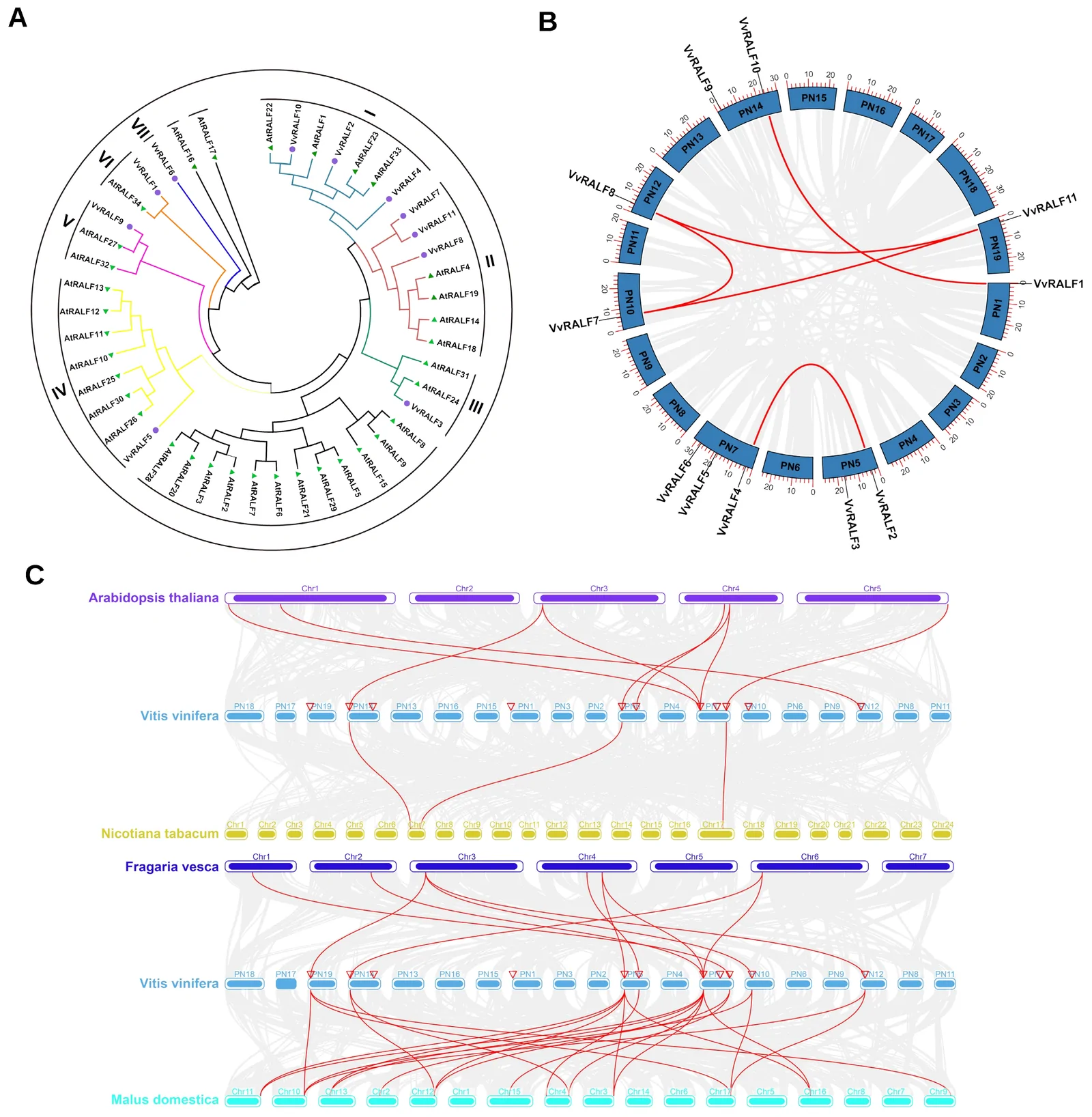
Phylogenetic and syntenic analyses of RALFs. (**A**) Phylogenetic tree illustrating the relationships between RALF proteins in grapevine and *Arabidopsis*. *Arabidopsis* RALF proteins are marked with green triangles, whereas grapevine RALF proteins are indicated by purple circles. (**B**) Distribution and duplication patterns of *VvRALFs* across chromosomes. Segmental duplication pairs are connected by red lines. (**C**) Syntenic analyses of *VvRALFs* vs. *RALFs* in *Arabidopsis*, *Nicotiana tabacum*, *Fragaria ananassa*, and *Malus domestica*. Orthologous pairs are connected by red lines.

A neighbor-joining (NJ) phylogenetic tree was constructed to explore the evolutionary relationships between the 34 AtRALFs and 11 VvRALFs, which revealed that VvRALFs could be classified into seven distinct groups (I–VII). The number of VvRALFs in groups I and II was three, whereas in groups III to VII, there was one VvRALF in each group (Figure 1A). This classification differed from the *Arabidopsis* system, in which RALF proteins were typically organized into six groups. *VvRALFs* were irregularly distributed across seven chromosomes of the grapevine genome. Specifically, one gene was mapped to chromosomes 1, 10, 12, and 19, two genes were located on chromosomes 5 and 14, and three genes were identified on chromosome 7. Notably, the number of *VvRALFs* located at the proximal or distal ends of the chromosomes far exceeded that found in the central regions (Figure 1B). We examined potential gene duplication events in grapevine (Figure 1B; Table S1). Sequence analysis identified no tandem duplications, whereas 63.64% of *VvRALFs* (seven genes) were involved in five segmental duplication pairs. We then analyzed homologous gene pairs between *V. vinifera* and *A. thaliana*, *N. tabacum*, *Fragaria ananassa*, and *M. domestica*, identifying 8, 3, 10, and 23 orthologous pairs, respectively (Figure 1C). These results indicate that *V. vinifera* is most closely related to *M. domestica*, whereas its synteny with *N. tabacum* is relatively weak.

### 2.2. Gene, Protein, and Promoter Structure Analyses of VvRALFs

**Figure 2:**
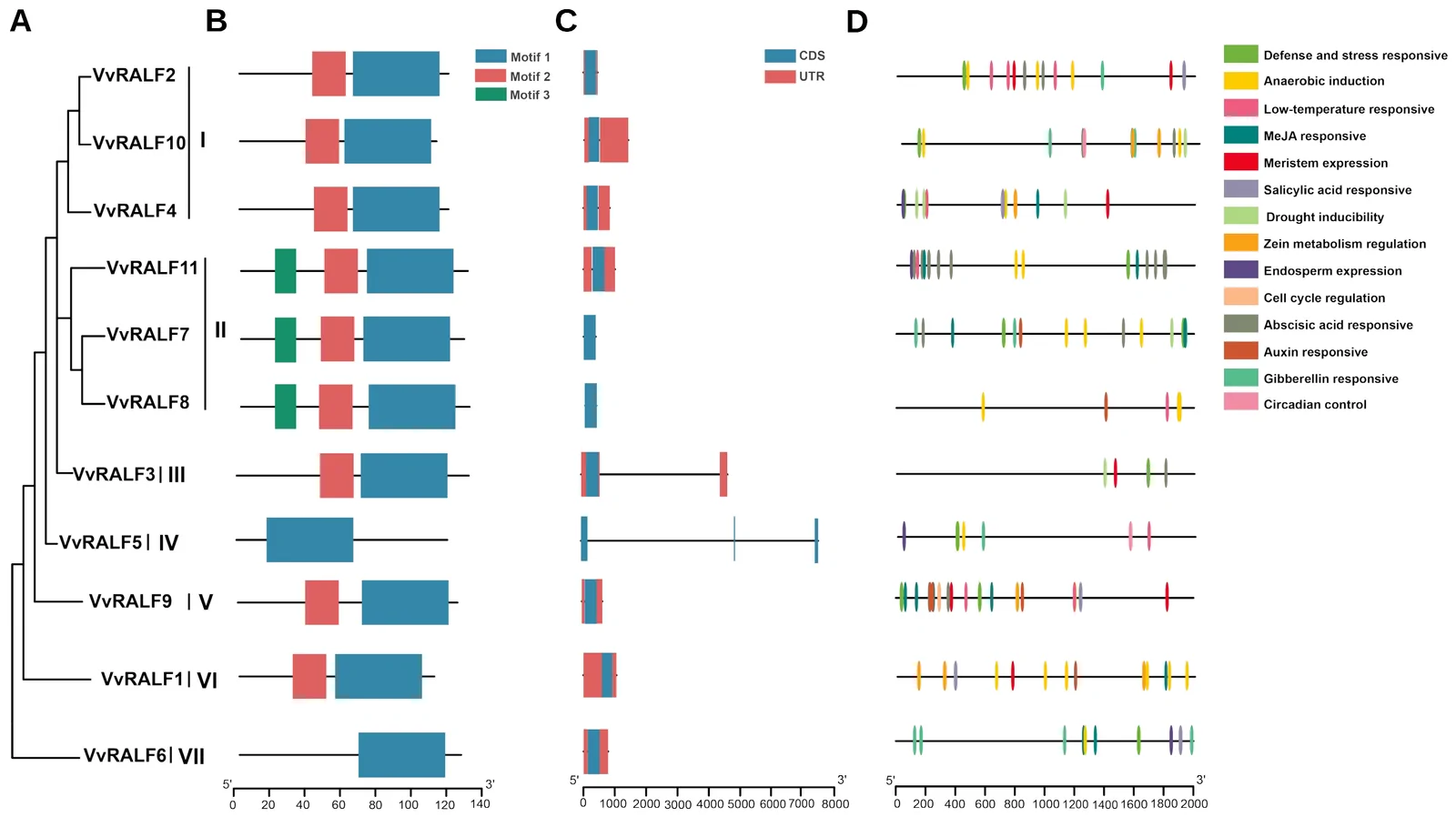
Gene, protein, and promoter structures of VvRALFs. (**A**) Phylogenetic relationships among VvRALFs. (**B**) Identification of conserved motifs in *VvRALFs*. Various motifs are represented by colored squares. (**C**) Gene structure of *VvRALFs* showing the distribution of UTRs (red squares) and coding sequences (CDSs) (blue squares) with introns denoted by black lines. (**D**) Major cis-regulatory elements within the *VvRALF* promoter. Various cis-elements are represented by colored squares.

The gene, protein, and promoter structures of *VvRALFs* were analyzed using GSDS 2.0, MEME, and PlantCARE, respectively. A phylogenetic tree, similar to that shown in Figure 1A, was constructed to associate these characteristics with evolutionary relationships (Figure 2A). Identification of conserved protein motifs is essential for understanding the evolutionary dynamics of gene families [27]. Three conserved motifs measuring 50, 20, and 13 amino acids in length were identified in VvRALFs. Notably, VvRALFs within the same phylogenetic group exhibited a consistent motif location and distribution. Specifically, groups I, III, V, and VI contained only motifs 1 and 2, whereas Group II included motifs 1, 2, and 3. The sequences in groups IV and VII contained only motif 1 (Figure 2B and Figure S2).

The exon–intron distribution of genes can significantly influence their functions [28]. To better understand this aspect, we analyzed the exon–intron organization of 11 *VvRALFs*, focusing on exon and intron number, length, and position. Genes within the same subfamily showed comparable structural characteristics, supporting the classification of the *VvRALFs*. Notably, all groups except group IV contained genes with a single exon, whereas group IV uniquely featured genes with three exons. Additionally, apart from the two genes in groups II and IV that lacked untranslated regions (UTRs), all other genes possessed both 5′- and 3′-UTRs. These structural variations could contribute to functional differences among the genes (Figure 2C).

Identifying the cis-regulatory elements of *VvRALFs* is crucial for predicting their transcriptional regulation and potential biological functions [29]. Six hormone-related cis-elements responsive to methyl jasmonate (MeJA), SA, ABA, auxin, and GA were identified, along with four putative stress-related and five growth-related cis-elements. Among the seven phylogenetic groups, the predominant cis-acting elements included those responsive to MeJA and ABA, as well as elements associated with meristem expression and zein metabolism regulation. Notably, the frequency and type of cis-regulatory elements related to hormones, stress responses, and plant growth varied between groups. Group I contained the highest abundance of elements related to plant growth and stress responses, whereas group II contained the highest abundance of hormone-related elements. These differences suggest that *VvRALFs* perform diverse biological functions that are influenced by variations in their cis-regulatory element profiles (Figure 2D and Figure S3).

### 2.3. Expression Patterns of VvRALFs in Different Tissues and Under Salt Stress

**Figure 3:**
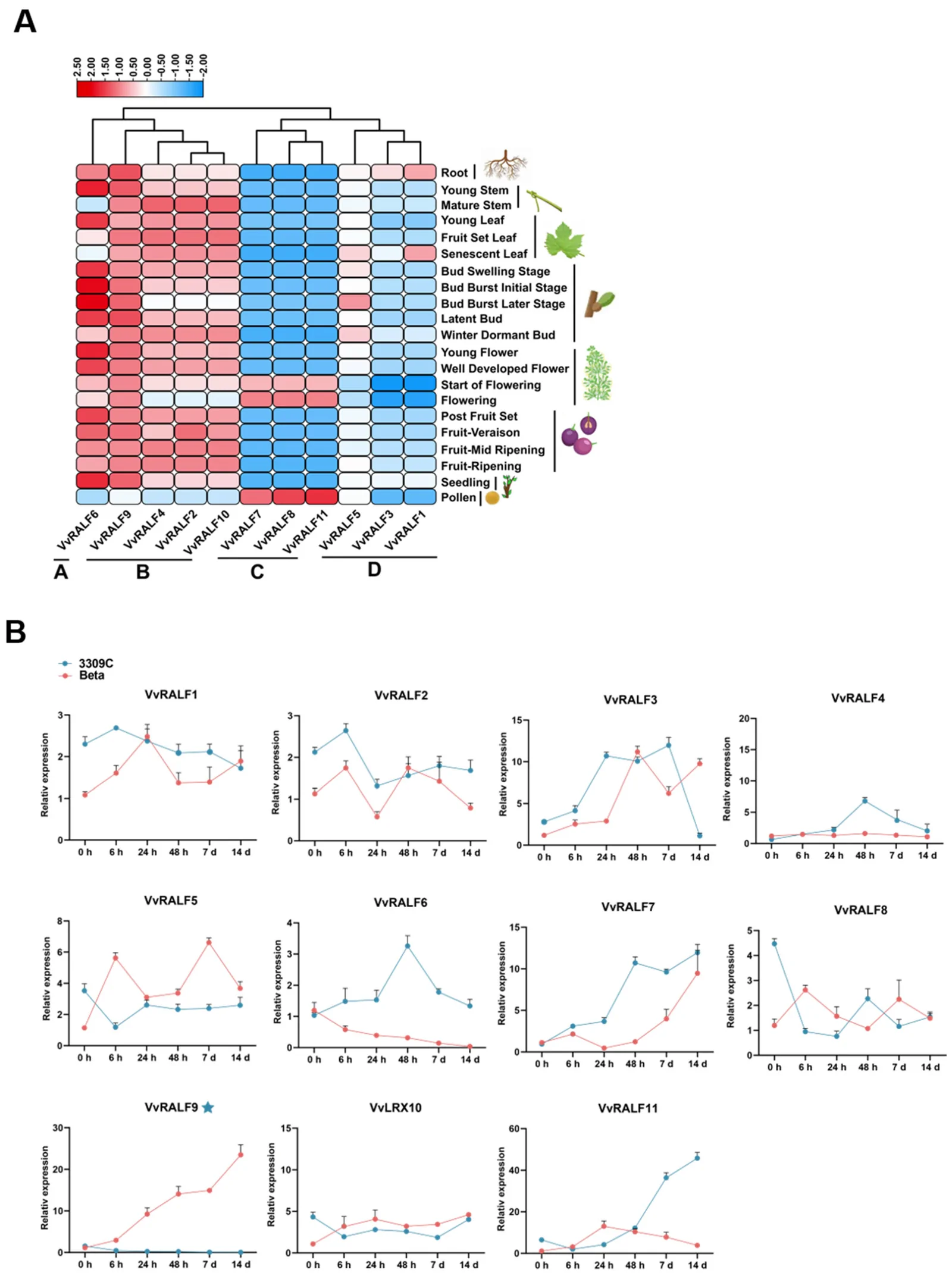
Expression profiles of *VvRALFs* in various tissues and under salt stress. (**A**) Tissue-specific expression of *VvRALFs*. A heatmap was generated using FPKM data obtained from the BAR database and visualized using TBtools-II with the “Row Scale” option. Expression levels are represented by a color gradient ranging from blue (low expression) to red (high expression). (**B**) Temporal expression patterns of 11 *VvRALFs* in the roots of ‘Beta’ and ‘3309C’ rootstocks after exposure to salt treatment. Error bars = standard deviation (SD).

To further explore the functions of *VvRALFs*, we analyzed their expression levels in 21 grapevine tissues and organs using data from the BAR database (Figure 3A). *VvRALFs* were broadly expressed across nearly all assessed tissues, and their expression profiles were classified into four major groups. Group A contained one gene with relatively high expression in all tissues except for the mature stem and pollen. Group B, comprising four genes, showed elevated expression in most tissues, excluding pollen. Group C included three genes that exhibited reduced expression across all tissues, except during flowering and pollen. Group D also contained three genes that exhibited reduced expression in all tissues, except the roots. These findings suggest that *VvRALFs* from different subgroups may play distinct roles in growth, development, and stress responses. To explore the potential involvement of *VvRALFs* in salt tolerance, we examined their expression in the roots of two grape rootstocks with differing levels of salt tolerance: the salt-sensitive ‘Beta’ and the salt-tolerant ‘3309C’ [30] at 6, 24, and 48 h, as well as 7 and 14 d following salt treatment (Figure 3B). Our results demonstrate that *VvRALF9* is the only member of the *VvRALF* family whose expression pattern correlates with rootstock salt resistance. It was consistently upregulated in the roots of salt-sensitive ‘Beta’ but downregulated in salt-tolerant ‘3309C’ following salt treatment. Given its inverse correlation with salt tolerance and high root-specific expression, *VvRALF9* was selected for further functional characterization.

### 2.4. VvRALF9 Negatively Regulates the Salt Tolerance of Grape Rootstock Roots

**Figure 4:**
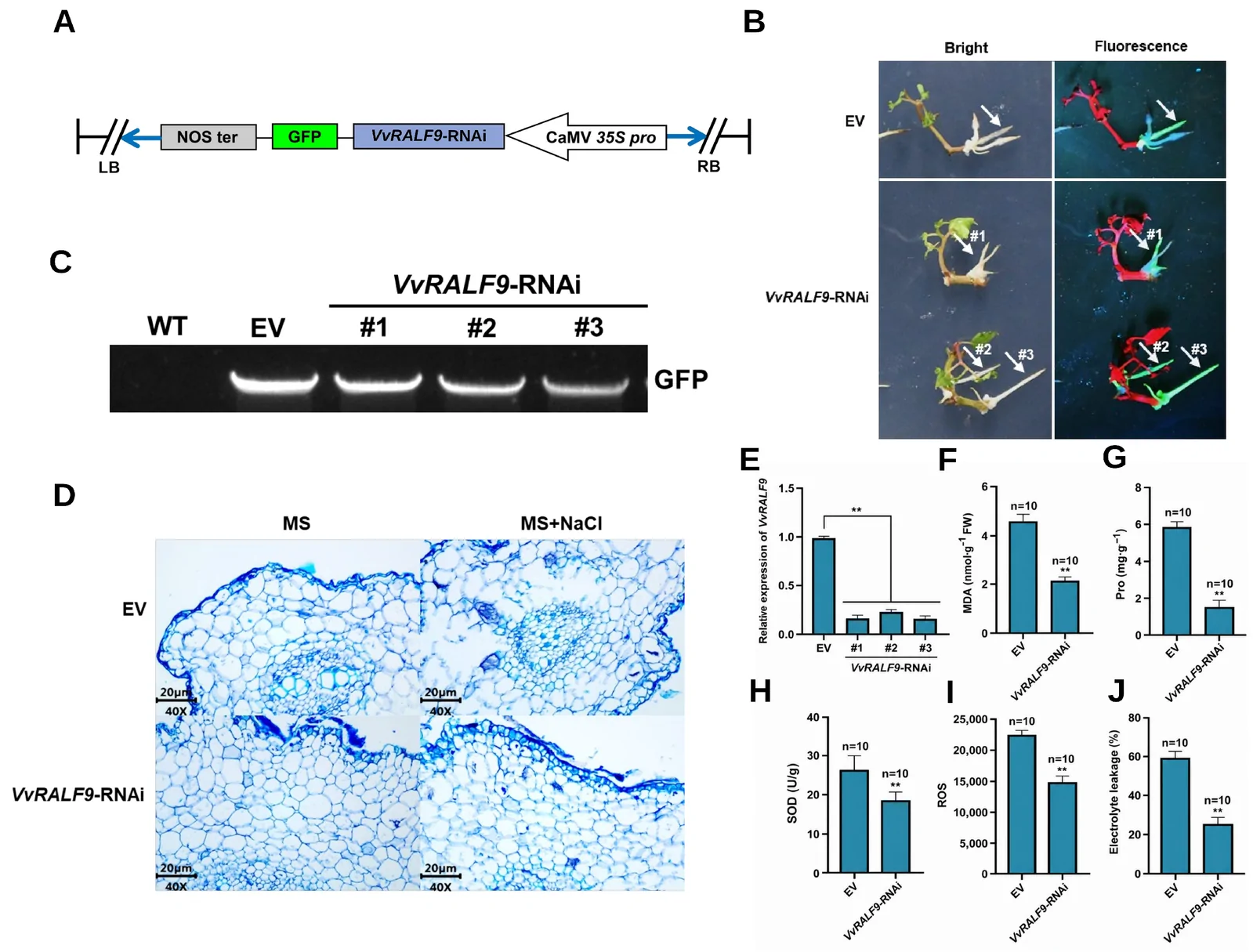
Enhancement of salt tolerance in grapevine rootstock roots through interference with *VvRALF9* expression. (**A**) Schematic representation of constructs used for *VvRALF9* RNAi. (**B**) Development of transgenic roots on wild-type (WT) ‘Beta’ shoots following infection with *R. rhizogenes*, along with GFP expression in transgenic grapevine roots under 480 nm light. Scale bar = 50 μm. (**C**) PCR confirmation of GFP-positive *VvRALF9*-RNAi hairy roots. (**D**) Histological structure of transgenic grapevine roots under salt treatment. Root sections were stained with toluidine blue and observed under a light microscope at 400× magnification. Scale bars = 20 μm. (**E**) Relative expression levels of *VvRALF9* in three independent *VvRALF9*-RNAi hairy-root lines. (**F**–**J**): (**F**) Malondialdehyde (MDA) content, (**G**) proline (Pro) content, (**H**) superoxide dismutase (SOD) activity, (**I**) reactive oxygen species (ROS) accumulation, and (**J**) relative electrolyte leakage (EL) in EV and *VvRALF9*-RNAi hairy roots after salt treatment. Data in (**F**–**J**) are presented as the means of 10 independent transgenic hairy roots, and error bars indicate the standard deviation (*n* = 10). Statistical significance relative to EV was determined using the Student’s *t*-test: ** *p* < 0.01.

The *VvRALF9* RNA interference (RNAi) fragment was cloned into the pROKII vector containing GFP driven by the CaMV 35S promoter (Figure 4A). *VvRALF9*-RNAi and empty vector (EV) transgenic roots were generated using a *Rhizobium rhizogenes*-mediated hairy-root transformation system. Independent positive roots were screened based on GFP fluorescence signals detected in newly developed hairy roots near the wound sites (Figure 4B), and GFP-positive roots were further confirmed by PCR amplification of GFP (Figure 4C). Three independent *VvRALF9*-RNAi positive roots were randomly selected for qRT-PCR validation. *VvRALF9* transcript levels were significantly lower in all three *VvRALF9*-RNAi roots than in EV roots, confirming efficient silencing of *VvRALF9* (Figure 4E).

To evaluate the effect of *VvRALF9* silencing on salt tolerance in grapevine rootstock roots, independent GFP-positive *VvRALF9*-RNAi and EV roots were subjected to 200 mmol·L^−1^ NaCl treatment. After 14 d of treatment, 10 independent transgenic roots from each group were used as biological replicates (*n* = 10) for histological observation and determination of physiological parameters. Salt treatment caused varying degrees of structural damage in both EV and *VvRALF9*-RNAi roots; however, the cellular and tissue structures of *VvRALF9*-RNAi roots remained relatively intact, with less pronounced damage than that observed in EV roots (Figure 4D). In EV roots, the malondialdehyde (MDA) and proline (Pro) contents, superoxide dismutase (SOD) activity, ROS fluorescence intensity, and relative electrolyte leakage (EL) were 4.59 nmol·g^−1^, 5.85 ng·g^−1^, 26.17 U·g^−1^, 22,674.46, and 58.98%, respectively. The corresponding values in *VvRALF9*-RNAi roots were 2.14 nmol·g^−1^, 1.53 ng·g^−1^, 18.33 U·g^−1^, 14,887.98, and 24.71%, respectively, all of which were significantly lower than those in EV roots (Figure 4F–J). These results indicate that silencing *VvRALF9* alleviates salt-induced ROS accumulation, membrane lipid peroxidation, and cellular membrane damage, thereby enhancing salt tolerance in grapevine rootstock roots.

### 2.5. VvFER2 Acts as a Potential Receptor of VvRALF9 in Grapevine and Positively Regulates Salt Stress Resistance of Grapevine Roots

**Figure 5:**
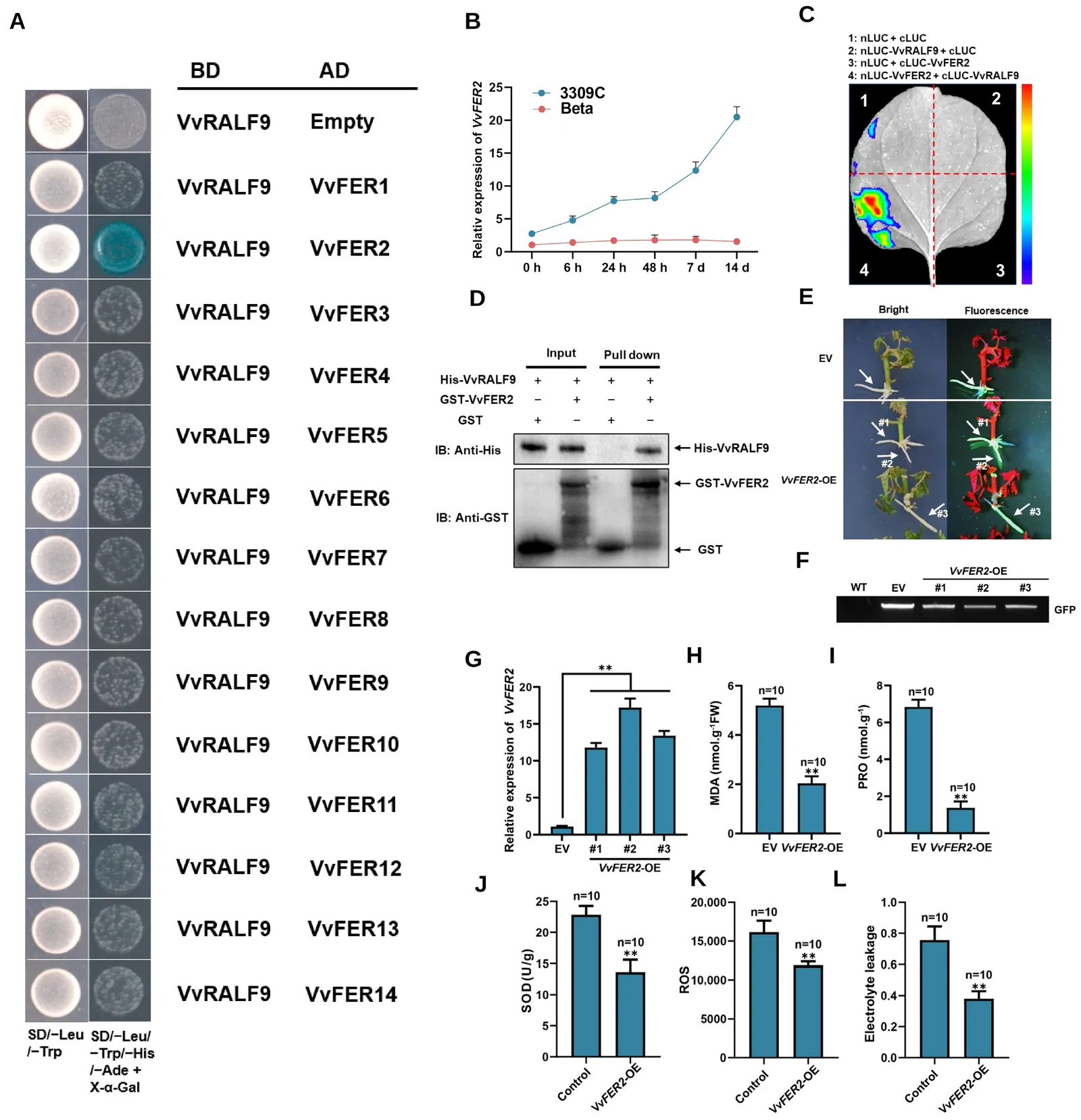
Interaction between VvRALF9 and VvFER2 and functional analysis of VvFER2 in salt tolerance. (**A**) Y2H testing of the interactions between VvRALF9 and VvFER1–14. (**B**) Expression patterns of *VvFER2* in the roots of ‘Beta’ and ‘3309C’ grapevine rootstocks were analyzed at different time points following salt treatment. Error bars = SD. (**C**) Split-luciferase complementation assay to confirm the interaction between VvRALF9 and VvFER2. (**D**) Pull-down assay demonstrating direct interaction between VvRALF9 and VvFER2. (**E**) Development of transgenic roots on WT ‘Beta’ shoots following infection with *R. rhizogenes*, along with GFP expression in transgenic grape roots under 480 nm light. Scale bar = 50 μm. (**F**) PCR confirmation of GFP-positive *VvFER2*-OE transgenic roots. (**G**) Relative expression levels of *VvFER2* in transgenic grapevine rootstock roots. (**H**) MDA content, (**I**) Pro content, (**J**) SOD activity, (**K**) ROS fluorescence intensity, and (**L**) relative electrolyte leakage (**E**,**L**) in transgenic roots under salt stress. Data in (**H**–**L**) are presented as the means of 10 independent transgenic hairy roots, and error bars indicate the standard deviation (*n* = 10). Statistical significance relative to EV was determined using the Student’s *t*-test: ** *p* < 0.01.

RALF peptides typically function through their receptor FER to regulate diverse aspects of plant growth, development, and stress responses [24,31]. In this study, a genome-wide analysis of *FERs* in grapevine identified 14 *VvFERs*. Yeast two-hybrid (Y2H) assays were conducted to investigate the interactions between VvFERs and VvRALF9, revealing that yeast cells co-expressing GAL4 DNA-binding domain (BD)-VvRALF9 and GAL4 activation domain (AD)-VvFER2 grew normally on −Trp/−Leu/−His/−Ade selective medium and turned blue, indicating a positive interaction (Figure 5A). Expression analysis indicated that *VvFER2* mRNA levels remained unchanged in the roots of the salt-sensitive rootstock ‘Beta’ but were upregulated in the salt-tolerant rootstock ‘3309C’ under salt stress (Figure 5B). To validate this interaction, a split-LUC assay was performed, in which strong luciferase activity was observed in tobacco leaves co-expressing N-terminal luciferase (nLUC)-VvRALF9 and C-terminal luciferase (cLUC)-VvFER2, confirming their interaction (Figure 5C). Further confirmation was provided by an in vitro pull-down assay, in which VvRALF9-His co-eluted with VvFER2-GST, supporting a direct physical interaction (Figure 5D). We then generated *VvFER2*-overexpressing (*VvFER2*-OE) and EV transgenic roots using the *R. rhizogenes*-mediated transformation system. Transgenic roots were detected using fluorescence-based screening around the wound sites (Figure 5E), and GFP-positive roots were further confirmed by PCR amplification of GFP (Figure 5F). qRT-PCR analyses demonstrated efficient overexpression of *VvFER2* in *VvFER2*-OE roots compared with EV roots (Figure 5G). To further assess the role of *VvFER2* in salt tolerance, MDA and Pro contents, SOD activity, ROS fluorescence intensity, and relative EL were measured in transgenic roots after exposure to 200 mM NaCl for 14 d (Figure 5H–L). Compared with EV roots, *VvFER2*-OE roots exhibited significantly lower MDA content (2.04 vs. 5.20 nmol·g^−1^), Pro content (1.38 vs. 6.85 ng·g^−1^), SOD activity (13.66 vs. 22.83 U·g^−1^), ROS fluorescence intensity (11,887.98 vs. 16,140.79), and relative EL (38.04% vs. 75.65%). These results further support a positive role for *VvFER2* in grapevine root salt tolerance.

### 2.6. Identification of Differentially Expressed Genes (DEGs) Between EV and VvRALF9-RNAi Roots

**Figure 6:**
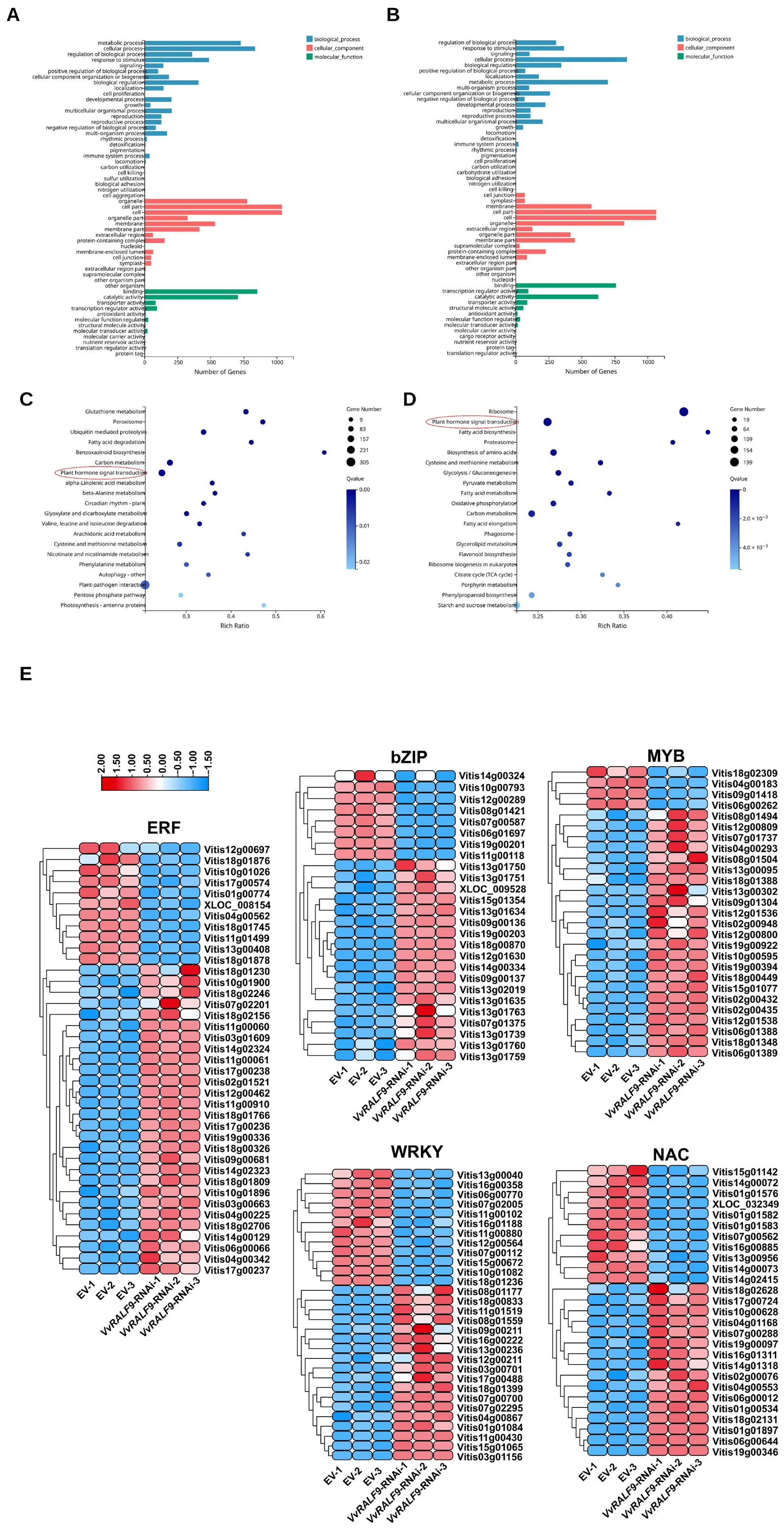
Identification of DEGs between the EV and *VvRALF9*-RNAi roots. GO classification of (**A**) upregulated and (**B**) downregulated DEGs in *VvRALF9*-RNAi vs. EV. KEGG pathway enrichment of (**C**) upregulated and (**D**) downregulated DEGs in *VvRALF9*-RNAi vs. EV. Only the top 15 terms with the lowest Q-value are shown. Red circles highlight the plant hormone signal transduction pathway. (**E**) Heatmaps of DEGs encoding salt tolerance-related TFs. Upregulation and downregulation are represented by red and blue, respectively.

To explore the transcriptional regulatory networks associated with VvRALF9 and obtain further insights into the potential regulatory mechanisms of the VvRALF9–VvFER2 module, RNA-Seq analysis was conducted to compare the gene expression between EV and *VvRALF9*-RNAi roots. A total of 2996 DEGs were identified, including 1549 upregulated and 1447 downregulated genes (Figure S4A). Notably, *VvRALF9* itself was identified as a DEG, confirming its successful downregulation in *VvRALF9*-RNAi roots compared to that in EV roots (log2 fold change (log2FC) = −2.7, false discovery rate (FDR) < 1.0 × 10^−12^) (Figure S4B). Gene Ontology (GO) classification and Kyoto Encyclopedia of Genes and Genomes (KEGG) pathway enrichment analyses were performed to examine the mechanisms underlying the functions of these DEGs. The majority of both upregulated and downregulated DEGs were primarily linked to the GO terms “cell part” and “cell” (Figure 6A,B), whereas KEGG analysis demonstrated notable enrichment in the “plant hormone signal transduction” pathway (Figure 6C,D).

Extensive transcriptomic studies across diverse plant species have shown that numerous TF families are essential for salt tolerance [26]. In this study, silencing *VvRALF9* by RNAi significantly altered the expression of multiple TFs (Figure 6E). Among them, the ERF family exhibited the largest number of DEGs, with 39 significantly up- or downregulated members, followed by the WRKY (30 DEGs), MYB (27 DEGs), NAC (27 DEGs), and bZIP (26 DEGs) families. Notably, across all TF families examined, more genes were upregulated than downregulated in response to *VvRALF9* silencing. Although ABA-dependent transcriptional regulation is largely mediated by the *AREB/ABF* subfamily of bZIPs [32,33], RNA levels of *AREB/ABF* genes did not differ between the *VvRALF9*-RNAi and EV roots, suggesting that these TFs may not contribute to the response to *VvRALF9* suppression under the conditions tested.

### 2.7. Silencing VvRALF9 Alters mRNA Levels of Hormone Signaling-Related Genes

**Figure 7:**
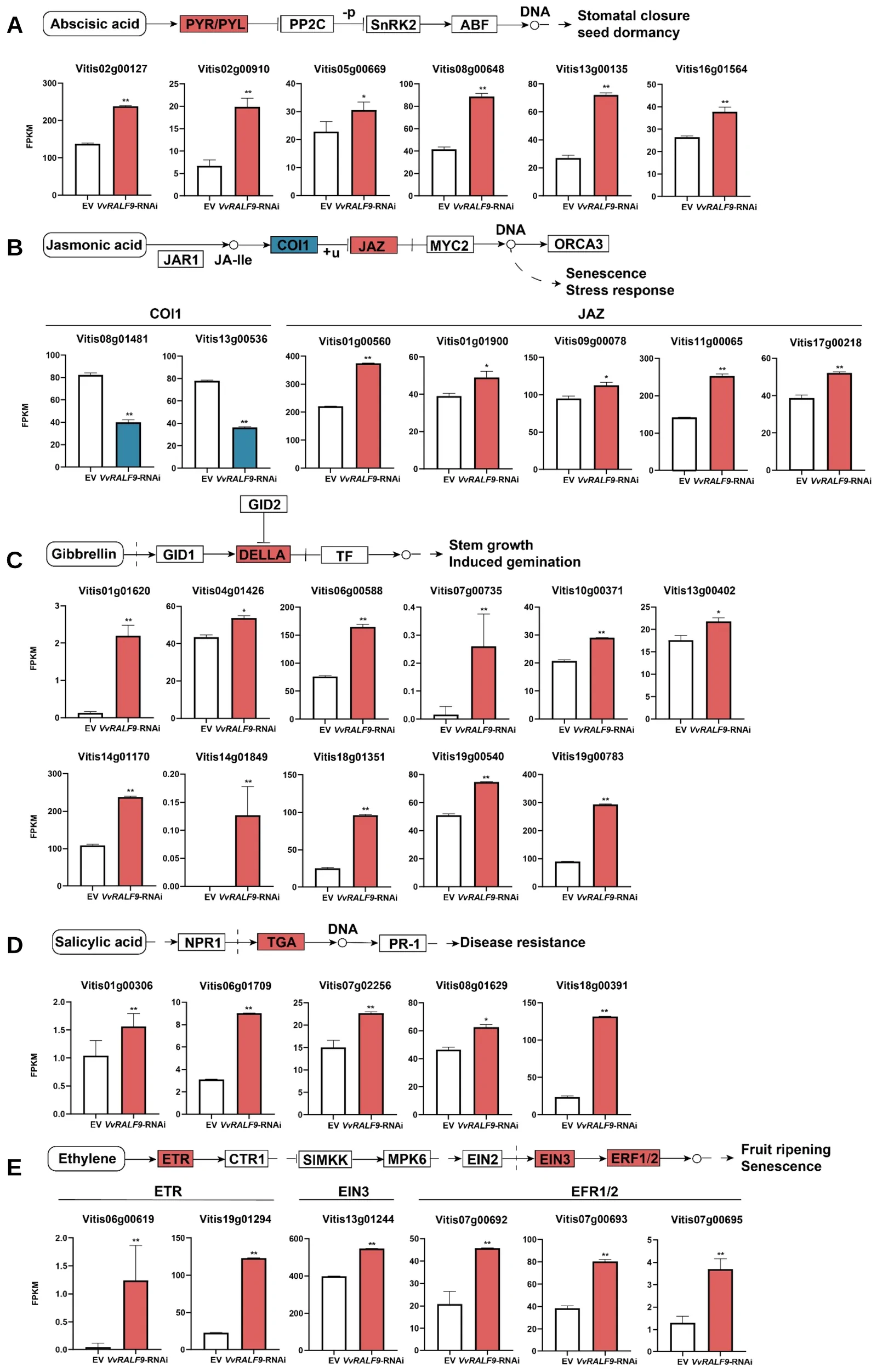
Expression of hormone signaling-related genes in the EV and *VvRALF9*-RNAi roots. (**A**) Six *PYR/PYL* genes related to ABA signaling were notably upregulated in *VvRALF9*-RNAi compared with EV. (**B**) Two *COI1* genes and five *JAZ* genes related to JA signaling pathways were notably downregulated and upregulated, respectively, in *VvRALF9*-RNAi compared with EV. (**C**) Eleven *DELLA* genes related to the GA signaling pathway were notably upregulated in *VvRALF9*-RNAi compared with EV. (**D**) Five *TGA* genes related to SA signaling were upregulated in *VvRALF9*-RNAi compared with EV. (**E**) Two *ETR* genes, one *EIN3* gene, and three *ERF1/2* genes related to ETH signaling were notably upregulated in the *VvRALF9*-RNAi roots compared with EV. Error bars = SD. Q values (adjusted *p*-values) were derived from DESeq2 (* Q < 0.05, ** Q < 0.01). Upregulation and downregulation are shown in red and blue, respectively.

Because KEGG analysis demonstrated notable enrichment in the “plant hormone signal transduction” pathway, we further analyzed the mRNA levels of hormone signaling-related genes. ABA signaling mediated by key regulators, such as *PYR/PYL*, PP2C, and SnRK2, plays a crucial role in salt stress response [34]. In this study, six *PYR/PYLs* were significantly upregulated in the *VvRALF9*-RNAi roots compared to the EV controls (Figure 7A). Similarly, JA serves as a major regulator of salt stress responses during specific plant growth stages, with *COI1* genes positively regulating JA signaling and *JAZ* genes negatively regulating it, both exhibiting differential expression [35]. Two *COI1* genes were downregulated in the *VvRALF9*-RNAi roots, whereas five *JAZ* genes exhibited increased expression (Figure 7B). Moreover, *DELLA* proteins involved in GA signaling enhanced plant survival by restricting growth under salt stress [36], and transcriptome analysis revealed 11 upregulated *DELLA* genes in the *VvRALF9*-RNAi roots (Figure 7C). *TGA* transcription factors essential for SA response could also contribute to salt stress tolerance, with five *TGA* genes found to be upregulated in the *VvRALF9*-RNAi roots (Figure 7D). Furthermore, mutations in ETH signaling genes, including *ETR*, *EIN3*, and *ERF1/2*, result in increased salt stress sensitivity [37]. Our data indicated significant upregulation of two *ETR* genes, one *EIN3* gene, and three *ERF1/2* genes in the *VvRALF9*-RNAi roots (Figure 7E). 

## 3. Discussion

Soil salinity severely restricts grapevine growth and production, yet the molecular mechanisms underlying salt adaptation in grapevine remain incompletely understood [38]. RALF peptides are important regulators of plant growth and stress responses [16,39], but their functions in grapevine have not been systematically investigated. In this study, we identified 11 *VvRALF* genes and characterized their evolutionary relationships, gene and protein structures, promoter features, and tissue-specific expression patterns. More importantly, salt-responsive expression analysis and functional assays identified VvRALF9 as a negative regulator of salt tolerance in grapevine roots and revealed VvFER2 as a potential interacting receptor associated with this response. Transcriptome analysis further indicated that *VvRALF9* suppression was accompanied by extensive changes in hormone signal transduction and stress-responsive transcriptional networks. Together, these results suggest that the VvRALF9–VvFER2 module participates in grapevine salt-stress responses through regulatory mechanisms involving hormone signaling and downstream transcriptional reprogramming.

Phylogenetic analysis classified the grapevine RALF family into seven groups (Figure 1A), showing both conserved and species-specific features compared with RALF families reported in other plants [13,17,18,19]. Notably, Group VII was specific to grapevine, whereas Groups I and II showed greater compositional variation than Groups III–VII, suggesting greater evolutionary diversification within these groups. The grapevine RALF family contained only 11 members, considerably fewer than the 34 reported in *Arabidopsis* [34]. Gene family expansion in plants is commonly associated with tandem and segmental duplication [40,41]; however, no tandem duplication events were detected among grapevine RALFs, whereas five pairs of segmentally duplicated genes were identified (Figure 1B and Table S1), which may partly account for the relatively small size of this family. Consistent with their phylogenetic relationships, members within several groups displayed similar gene structures and conserved motifs (Figure 2A–C and S2), whereas variation in promoter cis-acting elements (Figure 2D and Figure S3) and tissue-specific expression patterns (Figure 3A) suggested potential functional diversification among individual *VvRALFs* [13,18,42]. In particular, the presence of hormone-, development-, and stress-responsive elements in their promoters supports the possibility that different RALF members have been recruited into distinct regulatory processes in grapevine.

A particularly notable finding was that evolutionary proximity to AtRALF22/23 did not correspond directly to salt-responsive behavior in grapevine. In *Arabidopsis*, RALF22/23, together with LRX3/4/5 and FER, forms an important signaling module involved in salt-stress responses [22]. On the basis of evolutionary relatedness, grapevine RALFs most closely related to AtRALF22/23 might therefore be expected to retain similar salt-responsive functions. However, our results did not support such a simple relationship. *VvRALF10* showed the closest evolutionary relationship with AtRALF22/23, followed by *VvRALF2* and *VvRALF4* (Figure 1A), yet none of these genes responded significantly to salt stress in either the salt-sensitive rootstock ‘Beta’ or the salt-tolerant rootstock ‘3309C’, despite their relatively high transcript abundance in roots. In contrast, *VvRALF9*, which was less closely related to AtRALF22/23, was highly expressed in roots and exhibited strikingly contrasting responses to salinity, being upregulated in ‘Beta’ but downregulated in ‘3309C’ (Figure 3). This uncoupling between evolutionary relatedness and salt-responsive expression suggests that the recruitment of individual RALF family members into salt-stress regulation may have diverged between *Arabidopsis* and grapevine. Moreover, the opposite expression response of *VvRALF9* in rootstocks with contrasting salt tolerance points to a possible association between *VvRALF9* regulation and genotype-dependent adaptation to salinity.

Functional analysis further supported this interpretation. Under salt stress, silencing *VvRALF9* reduced ROS accumulation, MDA content, and EL, indicating the alleviation of oxidative and membrane damage in grapevine roots. Changes in proline accumulation and SOD activity further reflected altered physiological responses to salinity. Together with the contrasting expression patterns observed in ‘Beta’ and ‘3309C’, these results support a negative regulatory role for *VvRALF9* in grapevine salt tolerance (Figure 4). This finding is particularly relevant because it suggests that the functional contribution of grapevine RALFs to salinity cannot be inferred solely from sequence homology with *Arabidopsis* RALF22/23. Instead, species-specific and genotype-dependent regulatory divergence appears to be an important feature of RALF-mediated salt responses in grapevine.

RALF peptides commonly exert their biological functions through interactions with plasma membrane receptors, among which FER is one of the best characterized [20,21]. In *Arabidopsis*, FER contributes to root recovery under salt stress by sensing salt-induced changes in cell-wall integrity and activating downstream signaling [10]. In the present study, screening of grapevine FER proteins identified VvFER2 as a candidate interacting partner of VvRALF9, and this interaction was supported by Y2H, split-LUC, and pull-down assays (Figure 5A–D). Functional analysis further supported a positive role for VvFER2 in grapevine salt tolerance (Figure 5E–L). Under salt stress, the *VvFER2*-OE roots exhibited lower MDA and proline accumulation, together with reduced ROS accumulation and EL compared with the EV roots, indicating the alleviation of salt-induced oxidative and membrane damage. SOD activity was also lower in the *VvFER2*-OE roots; considering the simultaneous reduction in ROS accumulation, this pattern may reflect a lower oxidative burden and consequently a reduced requirement for antioxidant enzyme activity rather than impaired antioxidant capacity. Collectively, these physiological responses provide further evidence that *VvFER2* overexpression alleviates salt-induced cellular damage in grapevine roots. Thus, whereas *VvRALF9* negatively regulates salt tolerance, *VvFER2* is associated with enhanced salt resistance, supporting the involvement of a VvRALF9–VvFER2 signaling module in grapevine salt-stress responses. Nevertheless, although the present interaction assays demonstrate a physical association between VvRALF9 and VvFER2, further biochemical evidence will be required to define their precise ligand–receptor relationship and the molecular events linking VvRALF9 perception to VvFER2-dependent signaling.

A second important finding concerns the relationship between RALF–FER signaling and hormone regulation. In *Arabidopsis*, the RALF22/23–FER module has been associated with salt-stress responses through changes in the levels of JA, SA, and ABA [22]. In the present study, however, KEGG enrichment analysis of DEGs between the *VvRALF9*-RNAi and EV roots identified “plant hormone signal transduction” as a significantly enriched pathway, whereas pathways related to hormone biosynthesis and metabolism were not prominently enriched (Figure 6C,D). This distinction suggests that at the transcriptional level, the response associated with *VvRALF9* suppression in grapevine may be characterized primarily by the reprogramming of hormone perception and signal transduction rather than by broad changes in the transcription of hormone biosynthetic or metabolic pathways. Thus, although RALF–FER signaling is associated with hormone regulation in both *Arabidopsis* and grapevine, the regulatory level at which this connection is most strongly manifested may differ between species. This difference further supports the possibility that RALF–FER-mediated salt responses have undergone species-specific functional diversification.

Consistent with this interpretation, *VvRALF9* silencing affected multiple components of the ABA, JA, GA, SA, and ETH signaling pathways (Figure 7). ABA signaling is central to plant adaptation to osmotic and salt stress [2,34], and the increased expression of *PYR/PYL* genes in the *VvRALF9*-RNAi roots suggests altered regulation of ABA signaling components. In the JA pathway, downregulation of two *COI1* genes together with the upregulation of five *JAZ* genes indicated substantial reorganization of JA signaling, which is closely associated with root growth regulation under salinity [35,43]. Similarly, increased expression of *DELLA* genes is consistent with the growth-restraining role of GA signaling during stress adaptation [36], whereas altered expression of *TGA* genes suggests the participation of SA-associated signaling [44,45]. ETH signaling was also affected, as two *ETR* genes, one *EIN3* gene, and three *ERF1/2* genes were upregulated in the *VvRALF9*-RNAi roots [37,46]. Rather than indicating that these genes are direct targets of *VvRALF9*, these coordinated transcriptional changes suggest that suppression of *VvRALF9* is associated with broad reorganization of hormone-responsive signaling networks. In parallel, several major stress-responsive TF families, including WRKY, bZIP, ERF/AP2, MYB, and NAC, were differentially expressed (Figure 6E) [25,26]. These TFs may provide an additional regulatory layer linking altered hormone signaling to downstream stress-responsive gene expression.

**Figure 8:**
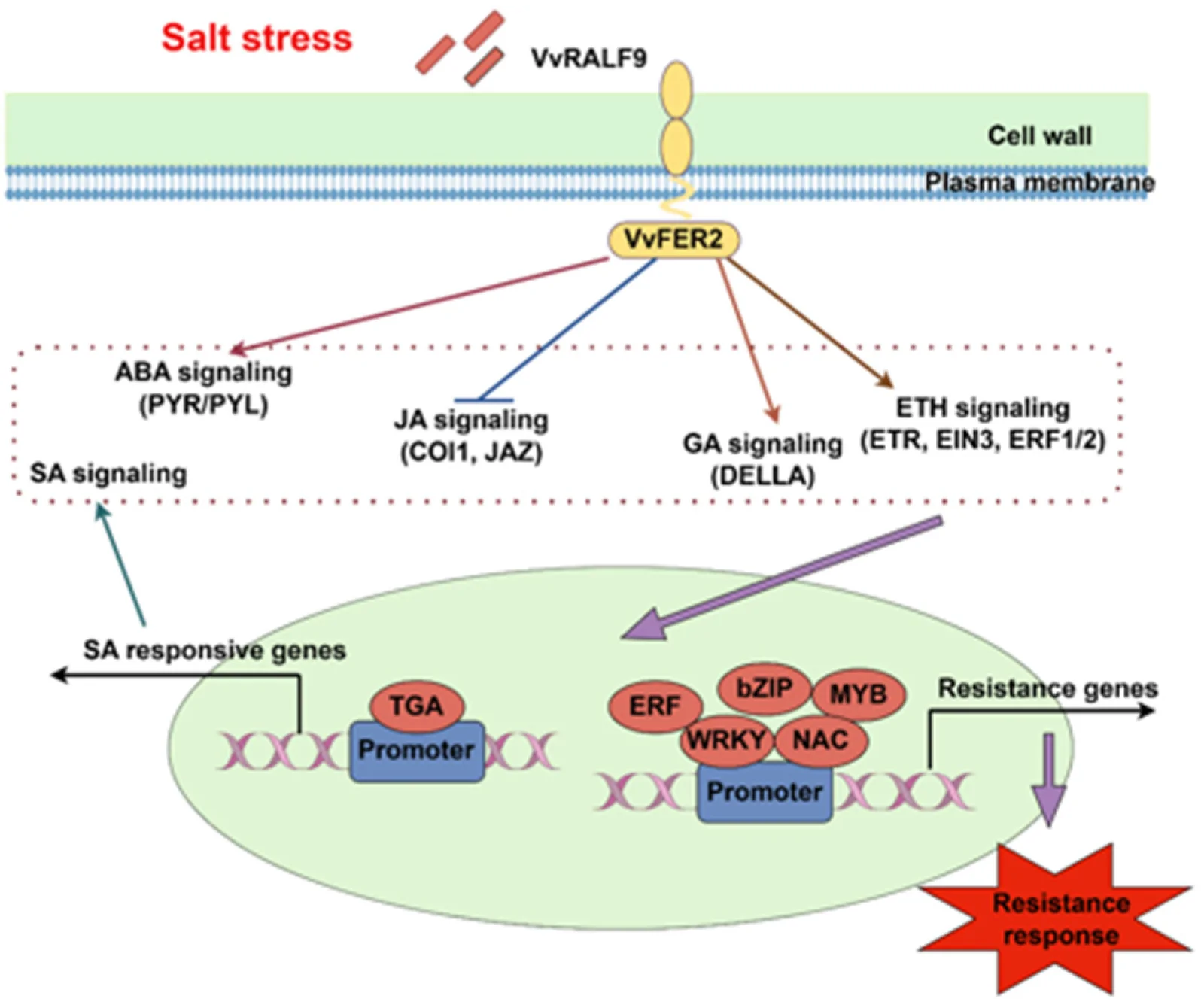
Proposed model of the VvRALF9–VvFER2 module regulatory pathway in grapevine roots under salt stress. Arrows indicate the proposed direction of signaling and regulatory interactions.

Taken together, our results support a working model in which the VvRALF9–VvFER2 module is involved in grapevine root responses to salinity (Figure 8). A notable feature of this model is that the salt-responsive RALF member identified in grapevine is not the closest homolog of Arabidopsis RALF22/23, indicating functional divergence of RALF-mediated salt regulation between the two species. Furthermore, suppression of *VvRALF9* was associated predominantly with transcriptional changes in hormone signal-transduction components and stress-responsive TFs, rather than with the prominent enrichment of hormone biosynthetic or metabolic pathways. These findings suggest that the VvRALF9–VvFER2 module may participate in coordinating hormone-responsive signaling and downstream transcriptional regulation during grapevine adaptation to salt stress. However, the direct molecular connections between VvFER2 and individual hormone-signaling components, as well as the extent to which these transcriptional changes reflect direct or indirect regulation, remain to be determined. Further studies integrating hormone measurements, receptor signaling analyses, and downstream target validation will be required to refine this regulatory model.

## 4. Materials and Methods

### 4.1. Genome-Wide Identification and Characterization of the RALF Gene Family in Grapevine

To identify RALF family members in grapevine, the protein sequences of 34 AtRALFs retrieved from the TAIR database (https://www.arabidopsis.org/browse/gene_family) (accessed on 19 December 2024) [47] were utilized as reference queries. BLASTP searches were performed against the Phytozome (https://phytozome-next.jgi.doe.gov/blast-search) (accessed on 22 December 2024) [48] and Winberige (http://www.winberige.cc/ftp.html) (accessed on 22 December 2024) databases using the PN_T2T genome of grapevine as a reference [49]. To ensure high-confidence identification, candidate sequences were filtered using an E-value threshold of <1 × 10^−30^. Candidate VvRALF sequences were further verified for the presence of RALF or RALF-superfamily domains using the NCBI-CDD (https://www.ncbi.nlm.nih.gov/Structure/cdd/wrpsb.cgi) (accessed on 9 April 2025) [50] and SMART (http://smart.embl.de/) (accessed on 9 April 2025) [51]. Sequences lacking the conserved RALF domain were excluded. Key physicochemical properties, including protein length, MW, and theoretical pI, were calculated using the ExPASy ProtParam platform (https://web.expasy.org/protparam/) (accessed on 11 April 2025) [52].

To investigate the evolutionary relationships and duplication patterns of VvRALFs, the full-length amino acid sequences of *Arabidopsis* and grapevine RALF proteins were aligned using ClustalW [53] implemented in MEGA X [54]. An unrooted NJ phylogenetic tree was subsequently constructed in MEGA X, and branch support was evaluated using 1000 bootstrap replicates. Chromosomal locations of *VvRALFs* were obtained from the Winberige database. Gene duplication and syntenic relationships were analyzed using MCScanX [55] with default parameters. Comparative synteny analyses were performed between *V. vinifera* and *A. thaliana*, *N. tabacum*, *F. ananassa*, and *M. domestica*. Chromosomal distributions and syntenic relationships were visualized using TBtools-II v2.310 [56].

To characterize the gene, protein, and promoter structures of *VvRALFs*, the gene structures of the 11 identified *VvRALFs* were analyzed using GSDS 2.0 (https://gsds.gao-lab.org/) [57] and visualized using TBtools-II v2.310 [56]. Conserved motifs in *VvRALF* proteins were identified using the MEME suite (https://meme-suite.org/meme/tools/meme) (accessed on 22 April 2025) [58], with motif widths ranging from 6 to 200 amino acids and the maximum number of motifs set to three. Promoter regions, defined as the 2-kb sequences upstream of the ATG of each *VvRALF* gene, were retrieved from the Winberige database using TBtools-II v2.310. Cis-regulatory elements within these promoter regions were annotated using the PlantCARE database (http://bioinformatics.psb.ugent.be/webtools/plantcare/html/) (accessed on 8 May 2025) [59] and visualized using TBtools-II v2.310.

To examine the tissue-specific expression patterns of *VvRALFs*, FPKM (fragments per kilobase of transcript per million mapped reads) data for *VvRALF* genes across 21 grapevine tissues and organs were retrieved directly from the BAR database (https://bar.utoronto.ca/) (accessed on 12 May 2025) [60]. The FPKM matrix was visualized as a heatmap using TBtools-II v2.310 with the “Row Scale” option enabled to facilitate comparison of the relative expression patterns of individual *VvRALF* genes across tissues.

### 4.2. Plant Materials, Growth Conditions, and Experimental Treatments

To investigate the salt-responsive expression patterns of *VvRALFs* and *VvFER2* in grapevine rootstocks, eighteen-month-old cutting-propagated ‘Beta’ (*Vitis riparia × Vitis labrusca*) and ‘3309C’ (*Vitis riparia × Vitis rupestris*) plants were grown in a greenhouse at the Shandong Academy of Grape (Jinan, Shandong, China). The greenhouse was maintained at daytime temperatures of 23–30 °C and nighttime temperatures of 6–9 °C, with a relative humidity of 55–65% and a 12-h photoperiod. Plants were cultivated in a growing medium consisting of substrate, vermiculite, and garden soil at a volume ratio of 3:2:1 [61].

For each rootstock, 15 plants with uniform growth were selected for salt-stress treatment. Five plants were pooled to constitute one biological replicate, and three biological replicates were included for each rootstock. Salt stress was imposed by a single irrigation with 200 mmol·L^−1^ NaCl solution until drainage was observed from the bottom of the pots [62]. Samples collected immediately before NaCl treatment (0 h) served as the untreated control. Newly developed root segments were collected at 0, 6, 24, and 48 h and at 7 and 14 d after salt treatment. For each biological replicate, root samples collected from five plants were pooled. All samples were immediately frozen in liquid nitrogen and stored at −80 °C until qRT-PCR analysis.

### 4.3. qRT-PCR Validation

To determine the expression levels of target genes in salt-treated and transgenic grapevine roots, total RNA was extracted from root samples using a commercial RNA extraction kit (TaKaRa Bio, Kusatsu, Japan) according to the manufacturer’s instructions. cDNA was synthesized from 1 μg of total RNA using the PrimeScript™ RT Master Mix (TaKaRa Bio, Kusatsu, Japan) according to the manufacturer’s protocol. Gene-specific primers were designed using SnapGene software (version 7.1.2; GraphPad Software, LLC, Boston, MA, USA) and are listed in Table S2.

qRT-PCR was performed using TB Green^®^ Premix DimerEraser™ (TaKaRa Bio, Kusatsu, Japan) on a CFX96™ Real-Time PCR Detection System (Bio-Rad Laboratories, Hercules, CA, USA). The amplification program consisted of an initial denaturation at 95 °C for 1 min, followed by 40 cycles of 95 °C for 5 s, 58 °C for 30 s, and 72 °C for 30 s. Each sample was analyzed with three technical replicates. *VvUBI* was used as the internal reference gene for normalization [29], and relative transcript levels were calculated using the 2^^−ΔΔCt^ method [63].

### 4.4. Vector Construction and Transgenesis

To investigate the functions of *VvRALF9* and *VvFER2* in grapevine salt tolerance, *VvRALF9*-RNAi and *VvFER2*-OE transgenic hairy roots were generated. The *VvRALF9* interference fragment was amplified from grapevine cDNA (Figure S5), whereas the CDS of *VvFER2* was amplified from cDNA prepared from ‘Beta’ roots. The *VvRALF9* interference fragment and *VvFER2* CDS were respectively inserted into the pROKII-GFP vector using the In-Fusion^®^ Snap Assembly Master Mix (TaKaRa Bio, Kusatsu, Japan). The resulting recombinant vectors, *VvRALF9*-RNAi-pROKII-GFP and *VvFER2*-pROKII-GFP, as well as the EV, were introduced into *R. rhizogenes* strain K599 using the freeze–thaw method. The cloning primers are listed in Table S2.

Shoot cuttings from ‘Beta’ grapevines, each containing at least two buds and leaves, were prepared under sterile conditions for transformation using *R. rhizogenes* [64]. The *VvRALF9*-RNAi-pROKII-GFP, *VvFER2*-pROKII-GFP, and EV constructs were separately introduced into the cuttings by co-cultivation with *R. rhizogenes* K599 in half-strength Murashige and Skoog (1/2 MS) liquid medium containing 50 μM acetosyringone. After 1 min of co-cultivation, the liquid medium was removed, and the explants were gently blot-dried and transferred to solid 1/2 MS medium for an additional 3 d of incubation at 23 °C in the dark. The explants were then transferred to fresh solid 1/2 MS medium supplemented with 300 mg·L^−1^ timentin to suppress bacterial growth and cultured at 26 °C under a 16-h light/8-h dark photoperiod. The medium was refreshed every 10 d until sufficient hairy roots had developed. Newly developed hairy roots were screened based on GFP fluorescence, and GFP-positive roots were further validated by qRT-PCR.

For salt-stress treatment, the transgenic hairy roots remained attached to the ‘Beta’ shoots and were transferred to solid 1/2 MS medium supplemented with 200 mmol·L^−1^ NaCl. After 14 d of salt treatment, GFP-positive *VvRALF9*-RNAi, *VvFER2*-OE, and EV hairy roots were collected for subsequent histological and physiological analyses.

### 4.5. Histological Analysis of Root Tissues

To evaluate salt-induced changes in root tissue structure following *VvRALF9* silencing, histological analysis was performed on EV and *VvRALF9*-RNAi hairy roots after 14 d of salt treatment. Root samples were collected and subjected to paraffin sectioning by Wuhan Maispu Biotechnology Co., Ltd. (Wuhan, China). Paraffin sections were prepared following a standard plant paraffin-sectioning protocol [65], including dehydration, paraffin infiltration, embedding, sectioning, staining, and mounting. Sections with a thickness of 8–10 μm were stained with toluidine blue to visualize tissue organization and cellular morphology. The stained sections were examined using an Olympus CX-21 light microscope (Olympus Corporation, Tokyo, Japan), and representative images were obtained at 400× magnification. Histological alterations in root tissue structure were compared between the EV and *VvRALF9*-RNAi roots.

### 4.6. Determination of Physiological Indicators

To assess the salt tolerance of transgenic grapevine rootstock roots, MDA and Pro contents, ROS accumulation, SOD activity, and relative EL were determined. MDA [66] and Pro [67] contents were quantified using the thiobarbituric acid reactive substances (TBARS) assay and the ninhydrin colorimetric method, respectively, according to a previously described method. ROS accumulation was determined using dihydroethidium (DHE) staining according to a previously established method [68]. Root segments were incubated in DHE staining solution at room temperature in the dark and subsequently washed with buffer to remove excess probe. The stained root tissues were observed using a confocal laser scanning microscope with excitation at approximately 488 nm, and the red fluorescence signals were recorded. ROS accumulation was evaluated by quantifying the fluorescence intensity of the acquired images and comparing the signals among treatments.

SOD activity was determined using the nitroblue tetrazolium (NBT) photochemical reduction method [69]. Root tissues were homogenized in phosphate buffer (pH 7.8), followed by centrifugation to obtain the crude enzyme extract. The reaction mixture contained the enzyme extract, methionine, NBT, EDTA-Na_2_, riboflavin, and phosphate buffer. The reaction was initiated by illumination, and absorbance was recorded at 560 nm. SOD activity was determined based on the inhibition of NBT photoreduction. Relative EL was measured according to a previously described method [70]. Root samples were frozen at −5 °C for 4 h and subsequently incubated in deionized water at 28 °C with shaking. The initial conductivity (C1) was recorded. After boiling and cooling to room temperature, the final conductivity (C2) was measured, and relative EL was calculated as (C1/C2) × 100%.

### 4.7. Y2H Assay

To screen for VvFER proteins that interact with VvRALF9, Y2H assays were performed according to a previously established protocol [71]. The CDS of *VvRALF9* was cloned into the pGBKT7 vector to generate the bait construct BD-VvRALF9, whereas the CDSs of *VvFER1–14* were individually cloned into the pGADT7 vector to generate the corresponding prey constructs. The primers used for vector construction are listed in Table S2. BD-VvRALF9 and each AD-VvFER construct were co-transformed into *Saccharomyces cerevisiae* strain Y2HGold. Transformants were first selected on SD/-Trp/-Leu medium for 2 d, after which positive colonies were transferred to SD/-Ade/-His/-Trp/-Leu medium and incubated for an additional 3 d. Protein–protein interactions were evaluated based on yeast growth and color development on the selective medium.

### 4.8. Split-LUC Assay

To validate the interaction between VvRALF9 and VvFER2 in planta, the CDSs of *VvRALF9* and *VvFER2* were fused with the nLUC and cLUC fragments, respectively, in the appropriate vectors. The primers used are listed in Table S2. The recombinant plasmids were introduced into *Agrobacterium tumefaciens* strain EHA105, and agroinfiltration was performed on tobacco leaves. Luminescence was captured using a PlantView100 plant live imaging system (Guangzhou Biolight Biotechnology Co., Ltd., Guangzhou, China) 48–60 h post-infiltration.

### 4.9. Protein Purification and Pull-Down Assay

To further validate the physical interaction between VvRALF9 and VvFER2 in vitro, the CDS of *VvRALF9* was inserted into the pET-32a vector to generate a His-tagged fusion protein, whereas the CDS of *VvFER2* was cloned into the pGEX4T-1 vector to generate a glutathione S-transferase (GST)-tagged fusion protein. Protein purification was performed as previously described [71], with VvRALF9-His and VvFER2-GST purified using Ni-NTA His·Bind Resin (7sea Biotech, Shanghai, China) and ProteinIso^®^ GST Resin (TransGen Biotech Co., Ltd., Beijing, China), respectively. The primers used for vector construction are listed in Table S2. The pull-down assay was performed using a Pierce™ GST Spin Purification Kit (Thermo Fisher Scientific, Rockford, IL, USA), and protein interactions were detected by Western blotting using anti-His and anti-GST antibodies (Abmart Shanghai Co., Ltd., Shanghai, China).

### 4.10. RNA-Seq Analysis

To investigate transcriptional changes associated with *VvRALF9* silencing, RNA-Seq analysis was performed using the EV and *VvRALF9*-RNAi roots, with three biological replicates for each group. Total RNA was extracted using the ethanol precipitation method combined with cetyltrimethylammonium bromide (CTAB)-PBIOZOL reagent. RNA sequencing was performed on the DNBSEQ platform (BGI-Shenzhen, Shenzhen, China) as previously described [72]. Raw reads were filtered using SOAPnuke v2.1.9 [73], and the resulting clean reads were aligned to the *Vitis vinifera* T2T reference genome using HISAT2 v2.2.1 [74]. Gene expression levels were quantified using RSEM v1.3.1 [75] and expressed as fragments per kilobase of transcript per million mapped reads (FPKM). DEGs between *VvRALF9*-RNAi and EV roots were identified using DESeq2 [76]. Genes with an absolute log2 fold change (|log2FC|) ≥ 1 and an adjusted *p*-value (Q value) ≤ 0.05 were considered differentially expressed. GO [77] classification and KEGG [78] pathway enrichment analyses were performed using the phyper function in R, followed by false discovery rate (FDR) correction.

## 5. Conclusions

In this study, we characterized the molecular features and expression patterns of 11 *VvRALFs* in grapevine. Our results support the involvement of the VvRALF9–VvFER2 module in grapevine salt stress responses and suggest that this module may be associated with changes in hormone signaling and transcriptional regulation. These findings provide new insights into the potential role of RALF–FER signaling in grapevine salt adaptation and offer a theoretical basis for the genetic improvement of salt-tolerant grapevine cultivars.

## Figures and Tables

**Table 1:** Characteristics of *VvRALFs*.

Name	ID	Location	Exon	Size (Amino Acids)	MW (kDa)	pI
*VvRALF1*	Vitis01g00024	PN1: 332,273–333,329	1	110	12.15	9.08
*VvRALF2*	Vitis05g00405	PN5: 3,392,998–3,393,840	1	118	12.91	8.99
*VvRALF3*	Vitis05g01287	PN5: 16,644,168–16,648,833	1	131	14.60	7.71
*VvRALF4*	Vitis07g00337	PN7: 3,297,514–3,297,968	1	118	12.66	7.54
*VvRALF5*	Vitis07g01360	PN7: 18,681,599–18,689,163	3	119	13.31	9.22
*VvRALF6*	Vitis07g02181	PN7: 27,384,759–27,385,540	1	125	14.32	8.58
*VvRALF7*	Vitis10g00736	PN10: 6,370,490–6,370,876	1	128	14.56	8.27
*VvRALF8*	Vitis12g00370	PN12: 4,252,824–4,253,213	1	129	14.52	7.72
*VvRALF9*	Vitis14g00238	PN14: 1,784,858–1,785,515	1	124	13.08	8.20
*VvRALF10*	Vitis14g01676	PN14: 23,525,341–23,526,745	1	111	12.34	8.96
*VvRALF11*	Vitis19g00266	PN19: 2,574,506–2,575,514	1	126	13.70	9.50

## Data Availability

Data are contained within the article and Supplementary Materials.

## Supplementary Materials

The following supporting information can be downloaded at: https://www.mdpi.com/article/10.3390/plants15213396/s1, Figure S1: Conserved domains analysis of *VvRALFs*; Figure S2: Sequence logos of 3 conserved motifs detected in the *VvRALFs*; Figure S3: Heatmap of the number of different cis-element types in *VvRALF* promoters; Figure S4: DEGs identified by transcriptome analysis by comparisons of *VvRALF9*-RNAi to EV roots; Figure S5: The sequence of *VvRALF9* interference; Table S1: The segmental duplication pairs of *VvRALF* genes in grapevine; Table S2: Primer sequences used in this study. F, forward primer; R, reverse primer.
