# Supplementary material for: VvRALF9–VvFER2 Module Mediates Grapevine Salt Stress Responses Through Hormone Signaling and Transcription Factor Regulation
Source: Plants (Basel). 2026 Nov 2;15(21):3396. doi: 10.3390/plants15213396 (PMC13766183; doi:10.3390/plants15213396)
Supplement: Supplementary file 1 [file plants-15-03396-s001.zip › plants-4536078-supplementary.pdf]

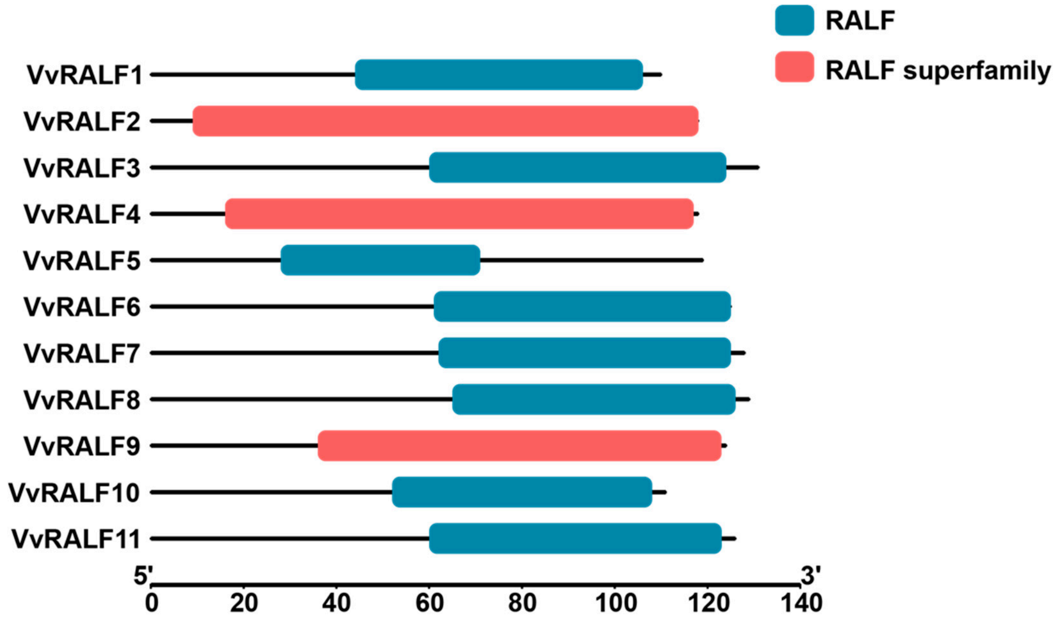

Figure S1. Conserved domains analysis of VvRALFs.

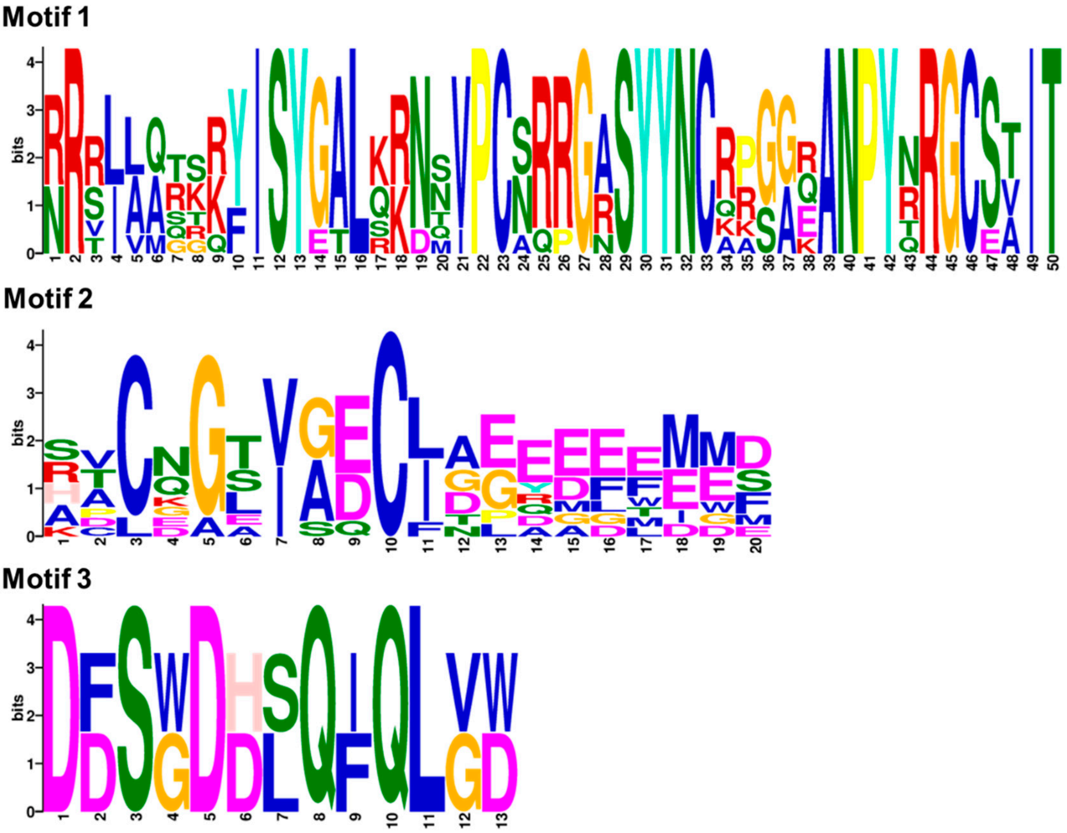

Figure S2. Sequence logos of 3 conserved motifs detected in the VvRALFs. The overall height of each stack represents the degree of conservation at this position, while the height of the individual letters within each stack indicates the relative frequency of the corresponding amino acid.

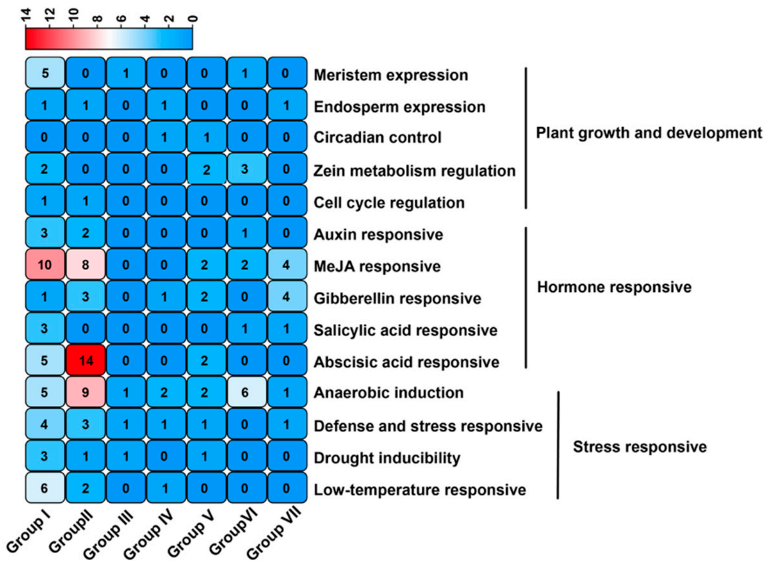

Figure S3. Heatmap of number of different cis-element types in *VvRALF* promoters. Red boxes indicate a higher number, whereas blue boxes indicate a lower number of each cis-element type.

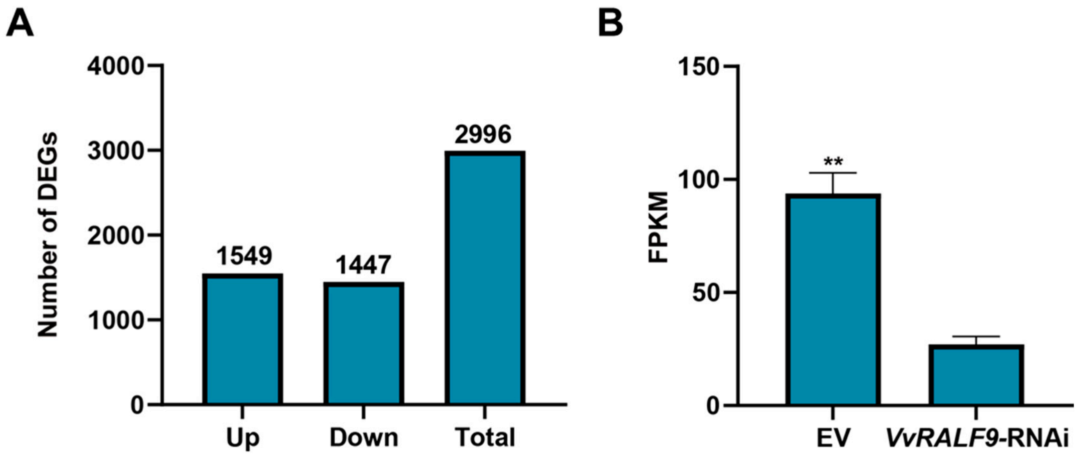

Figure S4. DEGs identified by transcriptome analysis by comparisons of *VvRALF9*-RNAi to EV roots. (A) The number of up- and down-regulated DEGs in *VvRALF9*-RNAi versus EV. (B) FPKM (fragments per kilobase of transcript per million mapped reads) values of *VvRALF9* in *VvRALF9*-RNAi and EV roots. Error bars represent standard deviation (SD). Statistical significance was assessed using Student's t-test (*P < 0.05, **P < 0.01).

ACTTCTGCAGCGAGTAATGGTGCTGCAACCACGGTTGTACGGATTTGCCTGAGCA
CCAGGCTGACAATTGTAGTAGGAAGCGCCACGTCGAGAGCACGGCACACTGTTC
CTCTGAAGCGCACCGTAGCTGATGTACTTGCTAGTCGCTAGGATGCGCCGGTTGA
TTTCCGAGTCCATCGCGAACTCTGAGGTCAGGCCGAACTCGTCTTC

Figure S5. The sequence of *VvRALF9* interference.

Table S1. The Segmental duplication pairs of VvRALF genes in grapevine

Segmental duplication pairs	
VvRALF1	VvRALF10
VvRALF2	VvRALF4
VvRALF7	VvRALF8
VvRALF7	VvRALF11
VvRALF8	VvRALF11

Table S2 Primer sequences used in this study. F, forward primer; R, reverse primer.

**Experiments**	**Primers**	**Sequence(5'→3')**
**qRT-PCR**		
VvRALF1	*VvRALF1*-qPCR-F	CAACACCAGCATACAGCAGC
	VvRALF1-qPCR-R	CTCATCCAACCAACTCCAGTCA
VvRALF2	*VvRALF2*-qPCR-F	AGAGATCGCCTTTCACTCTTGA
	VvRALF2-qPCR-R	CCGGAGTGTTTCCGAAGTCA
VvRALF3	*VvRALF3*-qPCR-F	TACTACAACTGCCGTGCCTC
	VvRALF3-qPCR-R	GTTGTTGCCCATCCACACAC
VvRALF4	*VvRALF4*-qPCR-F	ACGAGTTCGAGATGGACACG
	VvRALF4-qPCR-R	TAGTAGGACGCACCCCTCTG
VvRALF5	*VvRALF5*-qPCR-F	TCGACAACGGGGTTCTTGAC
	VvRALF5-qPCR-R	AGCACAAACAGGAGCCTCTC
VvRALF6	*VvRALF6*-qPCR-F	CCAGATGGGGTAATCGACCG
	VvRALF6-qPCR-R	TTGGAAGCAGTTGTGGGTGT
VvRALF7	*VvRALF7*-qPCR-F	CGGTGCAGTCGGTGATTGTA
	VvRALF7-qPCR-R	ACGGTTGCATGGTACTTGGT
VvRALF8	*VvRALF8*-qPCR-F	CGGTGCAGTCGGTGATTGTA
	VvRALF8-qPCR-R	ACGGTTGCATGGTACTTGGT
VvRALF9	*VvRALF9*-qPCR-F	ATCCGTCTGCAAGGGAACTG
	VvRALF9-qPCR-R	TTGCTAGTCGCTAGGATGCG
VvRALF10	*VvRALF10*-qPCR-F	CCTGGATACAATCGGCCCTG
	VvRALF10-qPCR-R	ACAAACAAACAGGGGGAAGATG
VvRALF11	*VvRALF11*-qPCR-F	CCCAAGGTGTTTAGTTCTGTGT
	VvRALF11-qPCR-R	TTGTTTGAGGAATGAGCCATCA
VvFER2	*VvFER2*-qPCR-F	ACCCATACCTCAAGTCCGAT
	VvFER2-qPCR-R	GGTCCATTTCCAGCATCTGA
VvUBI	*VvUBI*-qPCR-F	GTGGTATTATTGAGCCATCCTT
	VvUBI-qPCR-R	AACCTCCAATCCAGTTATCTAC
**Expression vector**		
VvRALF9-RNAi-pROKII-GFP	*VvRALF9-RNAi-pROKII-GFP-XbaI-*F	GAGAACACGGGGGACTCTAGAATGGCAAACTCCGTCGG

	VvRALF9-RNAi-pROKII-GFP-XbaI-R	ACCCTCGAGGGATCCTCTAGACTAACTTCTGCAGCGAGTAATGG
VvFER2-pROKII- GFP	*VvFER2-pROKII-GFP-XbaI*-F	GAGAACACGGGGGACTCTAGAATGAAGATGGAAAAGAAACATAGAGG
	VvFER2-pROKII-GFP-XbaI-R	ACCCTCGAGGGATCCTCTAGATTATCTACCACTGAGCCCAGAAAA
**Y2H**		
	VvRALF9-EcoRI-pGBKT7-F	ATGGCCATGGAGGCCGAATTCATGGCAAACTCCGTCGG
	VvRALF9-BamHI-pGBKT7-R	TGCGGCCGCTGCAGGTCGACCTAACTTCTGCAGCGAGTAATGG
	VvFER1-EcoRI-pGADT7-F	CCATGGAGGCCAGTGAATTCATGCTCACATTGTATCAGAGTAATTTTATTTA
	VvFER1-BamHI-pGADT7-R	CAGCTCGAGCTCGATGGATCCTTATCGGCCTTGGGGATTT
	VvFER2-EcoRI-pGADT7-F	CCATGGAGGCCAGTGAATTCATGAAGATGGAAAAGAAACATAGAGG
	VvFER2-BamHI-pGADT7-R	CAGCTCGAGCTCGATGGATCCTTATCTACCACTGAGCCCAGAAAA
	VvFER3-EcoRI-pGADT7-F	CCATGGAGGCCAGTGAATTCATGAATCTTGATTCCCATGTGTT
	VvFER3-BamHI-pGADT7-R	CAGCTCGAGCTCGATGGATCCTCATCTCCCTTTTGGGTTCA
	VvFER4-EcoRI-pGADT7-F	CCATGGAGGCCAGTGAATTCATGAGGCACAGCCCATGC
	VvFER4-BamHI-pGADT7-R	CAGCTCGAGCTCGATGGATCCTCATCTACCCTCAGACTTCACGAG
	VvFER5-EcoRI-pGADT7-F	CCATGGAGGCCAGTGAATTCATGCACATCAACAACACCCA
	VvFER5-BamHI-pGADT7-R	CAGCTCGAGCTCGATGGATCCTTATCGCCCTTTTGGTTTGAC
	VvFER6-EcoRI-pGADT7-F	CCATGGAGGCCAGTGAATTCATGAGGAATTTGTTTAAGTACCGTTG
	VvFER6-BamHI-pGADT7-R	CAGCTCGAGCTCGATGGATCCTCAACGTCCTTTTGGGTTCAT
	VvFER7-EcoRI-pGADT7-F	CCATGGAGGCCAGTGAATTCATGCCCACTTGTAGTAAAGGAAGC
	VvFER7-BamHI-pGADT7-R	CAGCTCGAGCTCGATGGATCCCTATGCATTGATTCCCCTTGTTG
	VvFER8-EcoRI-pGADT7-F	CCATGGAGGCCAGTGAATTCATGAGTTTGGATCAAATCAATCCC
	VvFER8-BamHI-pGADT7-R	CAGCTCGAGCTCGATGGATCCTCACCGTCCCTTTGGATTATTTAT
	VvFER9-EcoRI-pGADT7-F	CCATGGAGGCCAGTGAATTCATGACTGCTCGGATATTTCGAT
	VvFER9-BamHI-pGADT7-R	CAGCTCGAGCTCGATGGATCCTTATCGCCCGCTATGACTCC
	VvFER10-EcoRI-pGADT7-F	CCATGGAGGCCAGTGAATTCATGACTGTTCGGTTCTTTCGC
	VvFER10-BamHI-pGADT7-R	CAGCTCGAGCTCGATGGATCCTCAGAAAACATTCGCCGATG
	VvFER11-EcoRI-pGADT7-F	CCATGGAGGCCAGTGAATTCATGAGTTTGGATCAAATCAATCCC
	VvFER11-BamHI-pGADT7-R	CAGCTCGAGCTCGATGGATCCTCATCGTCCCTTTGTATTATCTATCTCA
	VvFER12-EcoRI-pGADT7-F	CCATGGAGGCCAGTGAATTCATGTTGCAGAAATCGGATTCTTT
	VvFER12-BamHI-pGADT7-R	CAGCTCGAGCTCGATGGATCCTCATACGCCAGAGGAATTGGT
	VvFER13-EcoRI-pGADT7-F	CCATGGAGGCCAGTGAATTCATGGATGACACCGGCATG

	VvFER13-BamHI-pGADT7-R	CAGCTCGAGCTCGATGGATCCTCATCGCCCCTTTGGG
	VvFER14-EcoRI-pGADT7-F	CCATGGAGGCCAGTGAATTCATGGAGAAAGTGAAATGGGGG
	VvFER14-BamHI-pGADT7-R	CAGCTCGAGCTCGATGGATCCTCATCTTCCACGAGGGTTTACTAG
**Protein Purification for Pull-down**		
	VvRALF9-EcoRI-pET-32a-F	ATGGCTGATATCGGATCCGAATTCATGGCAAACTCCGTCGG
	VvRALF9-Sal Ⅰ-pET-32a-R	GAGTGCGGCCGCAAGCTTGTCGACACTTCTGCAGCGAGTAATGG
	VvFER2- EcoRI-pGEX4T-1-F	CGTGGATCCCCGGAATTCATGAAGATGGAAAAGAAACATAGAGG
	VvFER2-XhoI-pGEX4T-1-R	ACGATGCGGCCGCTCGAGTCTACCACTGAGCCCAGAAAA
**Split-LUC**		
	VvRALF9-nLUC-KpnI-F	ACGGGGGACGAGCTCGGTACCATGGCAAACTCCGTCGG
	VvRALF9-nLUC-Sal-R	CGCGTACGAGATCTGCTAACTTCTGCAGCGAGTAATGG
	VvFER2-cluc-KpnI-F	ACGGGGGACGAGCTCGGTACCGCGATGAAGATGGAAAAGAAACATAGAGG
	VvFER2-cluc-SalI-R	CGCGTACGAGATCTGGTCGACTTATCTACCACTGAGCCCAGAAAA
